# The Development of Animal Nutrition and Metabolism and the Challenges of Our Time

**DOI:** 10.3389/fvets.2014.00023

**Published:** 2014-11-13

**Authors:** Toshiro Arai

**Affiliations:** ^1^Department of Basic Veterinary Medicine, School of Veterinary Medicine, Nippon Veterinary and Life Science University, Tokyo, Japan

**Keywords:** metabolic disorders, comparative nutrition, nutrigenomics, endocrinology, ruminology

Nutrition is defined as the process of providing and obtaining the food necessary for the health and growth of animals. Food nutrients are utilized as the main energy source by an animal via various processes, including digestion and absorption in the digestive tract, blood transport, and metabolism in the cells. Regulation of animal nutrition is associated with the functions of various tissues and organs in animals. New and evolving concepts in animal nutrition and metabolism present new research challenges that require interdisciplinary collaboration, a rethinking of traditional disciplinary boundaries, and adaptation of new research methodology. In this article, I will focus on three of these challenges – comparative nutrition, the relationship between immunology and nutritional disease, and nutrigenomics – describing the development of recent advances and promising areas for future research, including new treatments for metabolic disease.

## Comparative Nutrition and Metabolism

Studies on comparative animal nutrition begin with analysis of the processes of energy metabolism. Energy metabolism and imbalances in metabolic processes can induce various diseases in animals. Energy metabolism is the process of ATP production. In tumor cells, energy metabolism processes, including the pentose phosphate pathway and the malate aspartate shuttle, are accelerated remarkably (1), whereas, ATP production through glycolysis decreases greatly in the tissues of diabetic animals (2). Monitoring ATP production in tissues is an effective way to understand the health conditions of animals. Further, the relationship between energy metabolism, endocrinology, and immunology is key to understanding metabolic disorders in animals.

In recent years, lifestyle-related metabolic diseases, such as obesity, hyperlipidemia, and diabetes mellitus have increased in prevalence in dogs and cats, as in human beings (3, 4). As also seen in human beings, obesity in animals is caused by overeating and physical inactivity, and is a risk factor for various diseases involving energy metabolism. Obesity is defined as ectopic lipid accumulation. Recent evidence suggests that reduced lipid storage in the adipose tissue of obese animals contributes to ectopic lipid accumulation in non-adipose tissues, such as liver, skeletal muscle, and pancreas, where lipotoxicity can impair metabolic function (5). Excess calories and physical inactivity induce hyperglycemia followed by increased insulin secretion, which accelerates fatty acid synthesis via activation of transcription factors, such as sterol regulatory element binding protein (SREBP)-1c. Acceleration of fatty acid synthesis induces ectopic lipid accumulation and increases visceral fat accumulation (the state of obesity). These recent findings emphasize the importance of veterinary research in understanding the pathogenesis and prevention of metabolic disease and for developing effective treatment.

In contrast to the detrimental effects of lipid in obesity and diabetes, ectopic lipid accumulation can be highly valuable in animal nutrition, such as in the marbling of beef. Feeding a high amount of cereal grains, such as corn or barley (high-calorie foods), changes the color of carcass fat from yellowish to white, and increases the chance of obtaining a higher quality grade of meat. The common gene for fatty acid synthesis between monogastric animals (dogs and cats) and ruminants is SREBP-1c. Regulation of the SREBP gene prevents occurrence of obesity in dogs and cats, and induces marbling in beef cattle. Further, the nutritional characteristics of rumen fermentation in cattle differ from those in monogastric animals like dogs and cats. Different kinds of metabolic diseases also are observed between companion animals and food/agricultural animals. Comparative nutrition, therefore, is an important viewpoint in the study of animal nutrition and metabolism.

## Immunology and Metabolic Disease

As the digestive tract is one of the most important immune organs, and adipose tissue is the biggest endocrine organ in the animal body, immunology and endocrinology are indispensable viewpoints in the study of animal nutrition. In obesity, marked aberration of adipokine (adipocytokine) secretion in visceral fat together with an imbalance in the production of pro- and anti-inflammatory adipokines are critical in the development of various aspects of the metabolic syndrome, such as insulin resistance. Plasma hormone concentrations can accompany changes in the immune states of animals. Monocyte chemoattractant protein-1 (MCP-1) is secreted from white adipose tissue in obesity and contributes to tissue macrophage accumulation and insulin resistance by inducing a chronic inflammatory state (6). Adipose tissues in obese animals are altered to secrete other pro-inflammatory cytokines such as TNF-α, MCP-1, and IL-6 (7), which cause a persistent low-grade inflammation. Obesity also induces an imbalance in the immune reaction of animals. For example, vaccination is less effective in malnourished (emaciated) animals, which cannot produce sufficient amounts of immunoglobulins for healthy immunity. As in human beings, the relationship between nutrition (energy metabolism) and immunology is important for understanding the prevention and treatment of metabolic disorders in animals, including lifestyle-related diseases.

## Nutrigenomics

Effects of nutrients on gene expression have been studied well in recent years. Nutrigenomics, a branch of nutritional genomics, is the study of the effects of foods and food constituents on gene expression. Nutrigenomics has also been described as the influence of genetic variation on nutrition, where gene expression or single nucleotide polymorphisms (SNPs) are correlated with a nutrient’s absorption, metabolism, elimination, or biological effects. Nutrigenomics has recently emerged as an important viewpoint in animal nutrition. Nutrigenomics can lead to the development of effective foods for many diseases in animals, for instance, weight reducing diets that contain optimal constituents for obese animals. Metabolomics is the scientific study of chemical processes involving metabolites. Specifically, metabolomics is the “systemic study of the unique chemical fingerprints that specific cellular processes leave behind.” The metabolome represents the collection of all metabolites (the end products of cellular processes) in a biological cell, tissue, organ, or organism (8). Measurement of metabolites in animals with metabolic disorders can clarify the underlying cause of the disorder, such that metabolomics is very effective for the diagnosis of disease in animals. Ruminant metabolomics, the measurement of fermentation products in the rumen, is very important for investigation of health in ruminants. Exhaustive investigation of the effects of nutrients on plasma metabolite concentrations will be further developed as “nutrimetabolomics” for animals. Animal Nutrition and Metabolism encourages nutrigenomic studies for improvement of animal health and for development of new diagnostic and treatment methods for nutritional diseases.

## New Treatments for Metabolic Disorders

This brings us to a final challenge: developing promising treatments for metabolic disease based on our new understanding of the role of immunology. Early treatment of metabolic disorders includes the development of customized food, supplements, and drugs. Recently, good candidates of supplements and drugs for treating obesity in human medicine were developed, and these compounds will now be studied and utilized in veterinary medicine. Licorice flavonoids, for example, have antioxidative and anti-inflammatory activities and are very effective for treating obesity in animals (9). Apoptosis inhibitor of macrophage (AIM) is a macrophage-derived blood protein that plays a key role in the pathogenesis of atherosclerosis, metabolic diseases, and obesity-associated autoimmune diseases. The regulation of blood AIM levels or AIM function has the potential to serve as a next-generation therapy against these inflammatory diseases (10). AIM also may be a good candidate for treating metabolic disorders of animals through the new concept of drug development.

Animal nutrition and metabolism aims to publish research on new and effective drugs for various diseases of many animal species through analyses of energy metabolism using new analytical techniques, i.e., genomics, proteomics, and metabolomics. Energy metabolism is the origin of animal health; imbalances in energy metabolism lead to animal disease. Studies on animal nutrition and metabolism, therefore, will benefit the health conditions of various animals. Topics of interest include food (nutrients), malnutrition, vitamin deficiency, rumen fermentation, restricted feeding, inflammation, metabolic disorders, lifestyle-related illnesses (obesity, metabolic syndrome, diabetes, hypertension), tumors, genetic diagnosis, and development of supplements and drugs in animals.

While I cite only a few major challenges (comparative nutrition, nutrition–immunology relationship, nutrigenomics) in the immediate future of animal nutrition and metabolism, I know that there will be many others. With the support of the Editorial Board and the entire Animal Nutrition and Metabolism Community, Frontiers in Veterinary Sciences will be the major forum for addressing these and other future challenges. Welcome to the new journal and the new, innovative style of publishing.

## Conflict of Interest Statement

The author declares that the research was conducted in the absence of any commercial or financial relationships that could be construed as a potential conflict of interest.
